# Regional Spontaneous Neural Activity Alterations in Type 2 Diabetes Mellitus: A Meta-Analysis of Resting-State Functional MRI Studies

**DOI:** 10.3389/fnagi.2021.678359

**Published:** 2021-06-17

**Authors:** Jieke Liu, Yong Li, Xi Yang, Hao Xu, Jing Ren, Peng Zhou

**Affiliations:** Department of Radiology, Sichuan Cancer Hospital and Institute, Sichuan Cancer Center, School of Medicine, University of Electronic Science and Technology of China, Chengdu, China

**Keywords:** type 2 diabetes mellitus, resting-state functional magnetic resonance imaging, spontaneous neural activity, meta-analysis, seed-based *d* mapping

## Abstract

**Objective:** Resting-state functional magnetic resonance imaging (rs-fMRI) studies have revealed inconsistent regional spontaneous neural activity alterations in patients with type 2 diabetes mellitus (T2DM). The aim of our meta-analysis was to identify concordant regional spontaneous neural activity abnormalities in patients with T2DM.

**Methods:** A systematic search was conducted to identify voxel-based rs-fMRI studies comparing T2DM patients with healthy controls. The permutation of subject images seed-based *d* mapping (SDM) was used to quantitatively estimate the regional spontaneous neural activity abnormalities in patients with T2DM. Metaregression was conducted to examine the associations between clinical characteristics and functional alterations.

**Results:** A total of 16 studies with 19 datasets including 434 patients with T2DM and 391 healthy controls were included. Patients with T2DM showed hypoactivity in the right medial superior frontal gyrus, right superior temporal gyrus, and left lingual gyrus, whereas hyperactivity in the right cerebellum. Metaregression analysis identified negative correlation between regional activity in the medial superior frontal and anterior cingulate gyri and illness duration of patients with T2DM.

**Conclusion:** The patterns of regional spontaneous neural activity alterations, characterized by hypoactivity in the medial pre-frontal cortex, visual cortex, and superior temporal gyrus, whereas hyperactivity in the cerebellum, might represent the underlying neuropathological mechanisms of T2DM.

## Introduction

Type 2 diabetes mellitus (T2DM) is a chronic metabolic disorder characterized by chronic hyperglycemia, leading to long-term macrovascular and microvascular complications of various organ systems, including multiple central nervous system deficits, which are often associated with dementia, cognitive deterioration, and emotional dysfunctions (Kodl and Seaquist, 2008; Moheet et al., 2015; Thakur et al., 2019). Growing evidence does show that the cognitive decrements in T2DM patients appear to be attributable to brain structural and functional alterations (van Harten et al., 2006; Moran et al., 2013).

Magnetic resonance imaging (MRI) is a powerful tool to study the brain mechanism underlying the cognitive dysfunction of diabetes with quantitative measures. In recent decades, there is a growing interest of the use of resting-state functional MRI (rs-fMRI) to study the neurophysiological mechanism of T2DM because of its non-invasive and task-free nature. The amplitude of low-frequency fluctuation (ALFF)/fractional ALFF (fALFF) and regional homogeneity (ReHo) are two methods commonly used for characterizing local spontaneous neural activity of rs-fMRI data (Zuo and Xing, 2014). The ALFF in the blood oxygenation level–dependent signal has been correlated with local field potential activity (Logothetis et al., 2001), and the fALFF is strongly coupled with ALFF and quantifies the amplitude of these low-frequency oscillations (Zou et al., 2008). The ReHo is thought to reflect the similarity or synchronization between the time series of a given voxel and its nearest neighbors (Zang et al., 2004). In rs-fMRI studies, ALFF/fALFF and ReHo are usually used in “whole-brain voxel-wise” analysis with very similar preprocessing parameters across studies. Synthetically, the ALFF/fALFF and ReHo provide complementary information about the regional spontaneous brain activity (Xu et al., 2015).

Using the ALFF/fALFF and ReHo, regional spontaneous neural activity deficits in T2DM have been widely reported. However, they have produced inconsistent results. For example, increased regional activity has been found in the lingual gyrus, middle temporal gyrus, and precuneus (Wang et al., 2014; Zhou et al., 2014; Wang Z. L. et al., 2017; Liu Y. et al., 2020), whereas decreased activity in these regions has also been reported (Xia et al., 2013; Cui et al., 2014; Wang Y. F. et al., 2017), and yet more studies have identified no changes (Liu et al., 2016; Liao et al., 2019; Liu D. et al., 2020). This inconsistency resulted in part from small samples and from studying patients with various comorbidities, such as microangiopathy, including retinopathy, nephropathy, and peripheral neuropathy (Xia et al., 2013; Cui et al., 2014; Wang Z. L. et al., 2017; Liao et al., 2019; Qi et al., 2020); vitreous hemorrhage (Shi et al., 2020; Zhang Y. Q. et al., 2020); mild cognitive impairment (Zhou et al., 2014; Xiong et al., 2020); cirrhosis (Wang Y. F. et al., 2017); hypertension (Cui et al., 2014; Zhou et al., 2014); and dyslipidemia (Wang et al., 2014), which were known to alter brain function (Weissenborn et al., 2004; Umegaki et al., 2012; Umemura et al., 2013; Friedman et al., 2014; Muela et al., 2017). Thus, the central question of how the brain regional function manifests remains unaddressed in T2DM.

As a complex statistical method, meta-analysis involves the synthesis of data from relevant studies to identify an effect or draw a conclusion, and this approach can justify and refine hypotheses for various diseases (Mak et al., 2010). Comparing to the image-based meta-analysis, which requires the full image information, the peak probability meta-analysis methods such as seed-based *d* mapping (SDM, formerly Signed Differential Mapping) and activation likelihood estimation (ALE) are more feasible owing to they only using the peak coordinates. SDM adopted various positive features from previous methods such as ALE and multilevel kernel density analysis (MDKA), and introduced a series of improvements and novel features (Radua and Mataix-Cols, 2009). For example, SDM represents both positive and negative differences in the same map, thereby preventing a particular voxel from appearing to be significant in opposite directions (Radua et al., 2012). Another relevant feature is the use of effect sizes, which allows combination of reported peak coordinates with statistical parametric maps, thus allowing more exhaustive and accurate meta-analysis (Radua et al., 2012). Additionally, SDM enables several complementary analyses, such as subgroup and metaregression analyses, that can be used to assess the robustness and heterogeneity of the results (Radua et al., 2014). A previous meta-analysis in T2DM using ALE reported widespread brain function alterations including reduced activity in the lingual, postcentral, inferior temporal, cerebellar, insular, and posterior cingulate cortices as well as increased activity in the precuneus and superior frontal gyrus (Xia et al., 2017). However, this study involved functional connectivity measurements in addition to regional activity, and also included perfusion modality using arterial spin labeling (ASL).

Therefore, using a permutation of subject images seed-based *d* mapping (PSI-SDM) as primary tool, the aim of this study was to conduct a quantitative, voxel-based meta-analysis of whole-brain rs-fMRI studies in T2DM to investigate the regional spontaneous neural activity abnormalities and explore the potential effects of the clinical and demographic characteristics on these functional alterations. Although both ALFF/fALFF and ReHo measured the local activity of each voxel, previous studies found that ALFF and ReHo revealed convergent local activity alterations in some brain regions and also divergent brain regions with abnormal activity (Cui et al., 2014; Shi et al., 2020; Zhang Y. Q. et al., 2020). Hence, both combined and separate meta-analyses on ALFF/fALFF and ReHo were performed in this study.

## Methods

### Selection of Studies

A systematic search was conducted for relevant studies in the PubMed, Web of Knowledge, and EMBASE databases before December 31, 2020, according to PRISMA (Preferred Reporting Items for Systematic Reviews and Meta-Analyses) guidelines (Moher et al., 2010). The key search words were (“diabetes” or “diabetic”), (“amplitude of low-frequency fluctuation” or “ALFF” or “fALFF” or “regional homogeneity” or “ReHo”), and (“magnetic resonance” or “MRI” or “fMRI”). Besides, manual searches were conducted among the reference sections of the retrieved studies and suitable reviews. Studies were considered to be eligible according to the following criteria: (1) comparison of patients with T2DM with healthy controls; (2) using voxel-based analysis to investigate ALFF, fALFF, or ReHo changes in the entire brain or entire gray matter; (3) reporting of whole-brain results in a stereotactic space (Montreal Neurological Institute or Talairach); (4) published in English as an article. The corresponding authors were asked via email to send any additional data that were not included in the original publications. To minimize data entry error, all data from the initially retrieved studies were extracted by two radiologists. For each included study, we recorded the following: sample size, gender, age, education, illness duration, onset age, body mass index (BMI), hemoglobin A_1c_ (HbA_1c_)%, Mini Mental State Examination (MMSE) scores, scanner, acquisition parameters for rs-fMRI, software packages, and analytic methods.

### Voxel-Based Meta-Analysis

Voxel-based meta-analyses of regional brain differences were conducted with PSI-SDM software package (http://www.sdmproject.com, version 6.21). The procedures included the data preparation, preprocessing, mean analysis, and statistic test in brief.

In the data preparation, the peak coordinates and *t* values were written in a text file for each study. The studies with non–statistically significant unreported effects (NSUEs) were also included, and their text files were recorded with no content and named with the extension of “.no_peaks.txt” In the preprocessing, PSI-SDM first estimated the lower and upper bounds of possible effect-size images for the contrast between patients and controls from peak coordinates and effect sizes for each study separately. Second, PSI-SDM performed the meta-analysis of NSUE (MetaNSUE) based on maximum likelihood estimation and multiple imputation algorithm, which could include studies with NSUEs and was substantially less biased than previous versions of SDM. Briefly, PSI-SDM used the MetaNSUE to estimate the most likely effect size and its standard error and to create several imputations based on adding noise to these estimations within the bounds for each study (with or without NSUEs). Default number of imputations in PSI-SDM was used in this study (*n* = 50) (Radua et al., 2015; Albajes-Eizagirre et al., 2019a,b). In the mean analysis, PSI-SDM conducted a random-effects meta-analysis for each imputed dataset and then used Rubin's rules to combine the coefficients and their covariance and the heterogeneity statistics *I* and *Q* of these imputed datasets (Li et al., 1991; Radua et al., 2015; Albajes-Eizagirre et al., 2019a). In the statistical test, subject images were recreated in order to run a standard permutation test, in which the process was repeated with each set of permuted images. Statistical significance was determined by the maximum statistic from the images derived from the permuted images (voxel *p* < 0.005, peak height *z* = 1, cluster extent = 10 voxels) (Radua et al., 2012).

### Subgroup Analysis

To both establish consistency of findings and to identify measurements associated with divergent finding, we further performed secondary subgroup analyses to characterize brain regional function abnormalities within studies using ALFF/fALFF and ReHo separately.

### Metaregression Analysis

The potential effects of relevant demographic and clinical variables were examined by a random-effects general linear metaregression. The independent variables explored by the metaregression were mean age, percentage of males, education, illness duration, onset age, HbA_1c_%, BMI, and MMSE scores in patients with T2DM. The dependent variable was the SDM-*Z* value. The metaregression analyses were performed for studies using ALFF/fALFF and ReHo separately as well as their combinations. As reported in a previous study, we decreased the probability threshold to 0.0005 to reduce spurious results (Radua and Mataix-Cols, 2009). In the findings of whole-brain metaregression analysis, the regions that did not overlap with those in the main between-group analysis were discarded. Finally, regression plots were visually inspected to discard fittings driven by few studies (Radua and Mataix-Cols, 2009; Radua et al., 2012).

### Heterogeneity and Publication Bias Analyses

The between-studies heterogeneity of individual clusters was tested using a random-effects model. Magnitude of heterogeneity was estimated using *I*^2^ index, computed as 100% × (*Q* – *df*)/*Q*, where *df* is the degree of freedom, which estimates the proportion of variability due to non-random differences between studies. Funnel plots of significant clusters were also created by Egger tests (Egger et al., 1997).

## Results

### Included Studies and Sample Characteristics

**Figure 1:**
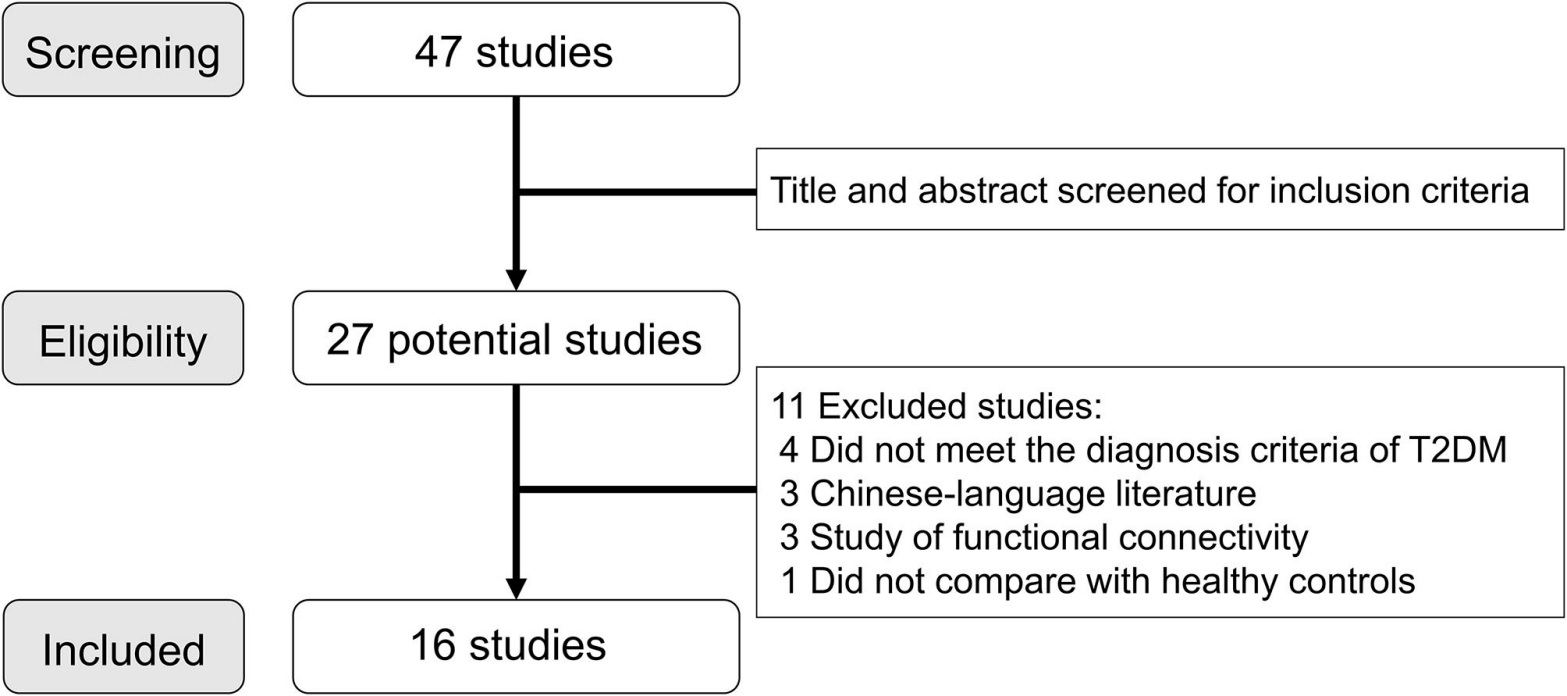
Literature search and study selection flowchart. T2DM, type 2 diabetes mellitus.

A total of 47 articles were identified through the systematic literature search, and Figure 1 shows the details of study selection process. We finally included 16 studies (19 T2DM datasets and 15 healthy controls datasets) in the final meta-analysis (Xia et al., 2013; Cui et al., 2014; Wang et al., 2014, 2019; Zhou et al., 2014; Liu et al., 2016; Peng et al., 2016; Wang Y. F. et al., 2017; Wang Z. L. et al., 2017; Liao et al., 2019; Liu D. et al., 2020; Liu Y. et al., 2020; Qi et al., 2020; Shi et al., 2020; Xiong et al., 2020; Zhang Y. Q. et al., 2020). In two studies of ReHo, the analysis was performed in two different T2DM subgroups (Peng et al., 2016; Xiong et al., 2020), and in another study, the analysis was performed with ALFF and ReHo (Cui et al., 2014). Besides, two studies used the same sample but analyzed two different measurements (Shi et al., 2020; Zhang Y. Q. et al., 2020). For these studies involving multiple independent patient subgroups or different measurements, the group coordinates were treated as separate datasets in the meta-analysis.

The search revealed 434 patients with T2DM and 391 healthy controls (Table 1). Acquisition parameters for rs-fMRI images, software packages, and analytic methods of all included studies are presented in the Supplementary Material. Patient groups were age- and sex-matched to control groups when combining the datasets. Specifically, the mean age was 56.7 ± 8.2 years old in the T2DM group vs. 55.8 ± 7.3 years old in the control group.

**Table 1:** Demographic and clinical characteristics of the included studies in the meta-analysis.

**References**	**Method**	**Patients with type 2 diabetes mellitus**									**Healthy controls**	
		**No. (% male)**	**Age (SD), years**	**Education(SD), years**	**Duration (SD), years**	**Onset(SD), years**	**BMI (SD), kg/m^**2**^**	**HbA_**1c**_%(SD)**	**MMSE (SD)**	**Comorbidity (no. of patients)**	**No. (% male)**	**Age (SD), years**
Xia et al. (2013)	ALFF	28 (53.6)	58.7 (8.1)	9.9 (3.7)	9.8 (5.5)	48.9 (NA)	25.4 (3.0)	7.9 (1.7)	NA	NA	29 (44.8)	57.7 (7.2)
Cui et al. (2014)	ALFF and ReHo	29 (48.3)	58.3 (7.3)	10.4 (4.0)	9.3 (3.8)	49.0 (NA)	NA	7.9 (1.7)	28.3 (1.4)	Retinopathy (8), peripheral neuropathy (8), lacunar infarcts (6)	27 (40.7)	57.8 (5.9)
Wang et al. (2014)	ALFF	26 (65.4)	54.7 (10.4)	11.2 (3.8)	NA	NA	25.9 (3.3)	8.3 (1.4)	27.8 (2.5)	Hypertension (10), dyslipidemia (10), lacunar infarcts (2)	26 (65.4)	54.9 (9.8)
Zhou et al. (2014)	ALFF	14 (42.9)	63.5 (6.9)	10.6 (2.7)	6.5 (2.1)	57.0 (NA)	24.8 (2.7)	7.8 (1.0)	25.1 (2.0)	MCI (14), hypertension (4)	17 (58.8)	63.8 (5.8)
Liu et al. (2016)	ReHo	25 (68.0)	52.2 (4.8)	11.0 (3.0)	7.7 (5.4)	44.5 (NA)	24.7 (3.2)	8.4 (NA)	27.9 (1.9)	NA	25 (52.0)	52.1 (3.5)
Peng et al. (2016)	ReHo	26 (46.2)	57.6 (9.3)	10.3 (2.9)	12.1 (5.8)	45.5 (NA)	24.3 (3.5)	8.8 (1.3)	28.8 (0.6)	Microangiopathy (26), hypertension (10), dyslipidemia (9)	28 (42.9)	56.2 (6.9)
	ReHo	22 (45.5)	58.8 (7.9)	10.0 (2.1)	10.9 (3.4)	47.9 (NA)	23.8 (3.2)	8.1 (2.2)	28.9 (0.7)	Hypertension (8), dyslipidemia (8)		
Wang Y. F. et al. (2017)	fALFF	17 (70.6)	54.8 (8.3)	NA	NA	NA	NA	NA	NA	Cirrhosis (17)	17 (70.6)	54.4 (7.9)
Wang Z. L. et al. (2017)	ALFF	21 (47.6)	54.9 (9.9)	NA	9.5 (5.0)	45.4 (NA)	NA	8.4 (1.7)	28.2 (1.1)	Retinopathy (21), nephropathy(4)	16 (56.2)	54.8 (5.7)
Liao et al. (2019)	ReHo	28 (42.9)	57.2 (5.2)	NA	NA	NA	NA	NA	NA	Retinopathy (28)	28 (42.9)	56.8 (5.1)
Wang et al. (2019)	ALFF	19 (42.1)	53.1(8.0)	NA	12.3 (5.3)	40.8 (NA)	NA	NA	NA	Retinopathy and nephropathy (19)	19 (42.1)	54.2 (9.0)
Liu D. et al. (2020)	ALFF	37 (64.9)	57.6 (7.1)	12 (NA)	NA	NA	25.1 (2.7)	7.4 (NA)	28 (NA)	NA	37 (45.9)	57.9 (5.7)
Liu Y. et al. (2020)	ReHo	26 (NA)	51.9 (10.7)	NA	NA	NA	24.0 (3.6)	NA	26.9 (3.9)	NA	26 (NA)	48.2 (6.7)
Qi et al. (2020)	ALFF	35 (51.4)	54.2 (8.7)	NA	9.9 (5.1)	41.5 (NA)	NA	7.5 (1.3)	NA	Retinopathy (35)	38 (47.4)	53.5 (7.7)
Xiong et al. (2020)	ReHo	25 (44)	62.7 (5.6)	11.1 (3.5)	9.0 (7.5)	53.7 (NA)	23.7 (2.9)	8.3 (1.6)	25.3 (2.0)	MCI (25)	27 (44.4)	59.1 (6.4)
	ReHo	25 (40)	59.0 (6.2)	11.6 (3.3)	5.7 (4.6)	53.4 (NA)	22.6 (2.5)	7.2 (1.4)	28.6 (1.0)	NA		
Shi et al. (2020)	ALFF	31 (51.6)	56.0 (4.6)	NA	27.2 (19.9)	NA	NA	5.3 (0.4)	NA	Vitreous hemorrhage (31)	31 (51.6)	56.5 (4.3)
Zhang Y. Q. et al. (2020)	ReHo											

*SD, standard deviation; BMI, body mass index; MMSE, Mini Mental State Examination; ALFF, amplitude of low-frequency fluctuations; fALFF, fractional ALFF; ReHo, regional homogeneity; MCI, mild cognitive impairment; NA, not available*.

### PSI-SDM Meta-Analysis

**Figure 2:**
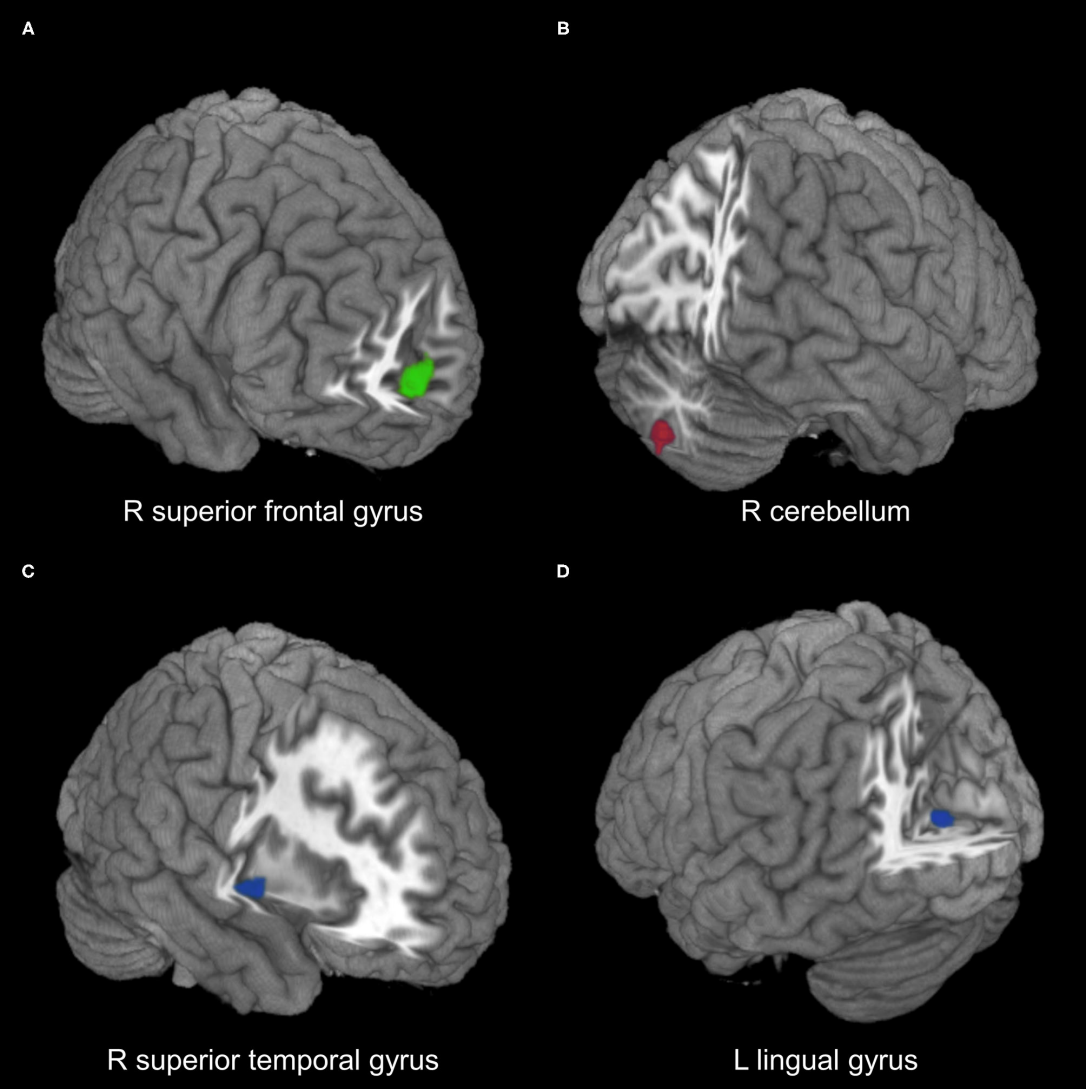
Meta-analysis results of regions with resting-state function alterations in type 2 diabetes mellitus relative to healthy controls. **(A)** Hypoactivity (green) in combined ALFF/fALFF and ReHo datasets. **(B)** Hyperactivity (red) in ALFF/fALFF datasets. **(C,D)** Hypoactivity (blue) in ReHo datasets. ALFF, amplitude of low-frequency fluctuations; fALFF, fractional ALFF; ReHo, regional homogeneity; R, right; L, left.

Coordinates for the meta-analyses were obtained from 18 datasets, and only one dataset had NSUE (Xiong et al., 2020). Patients with T2DM showed significant regional hypoactivity in the right medial superior frontal gyrus compared with healthy controls (Table 2, Figure 2). No significant regional hyperactivity was found between patients with T2DM and healthy controls.

**Table 2:** Meta-analysis results for patients with T2DM relative to healthy controls.

	**MNI coordinates**	**SDM-*Z* value**	***p*-value**	**No. of voxels**	**Cluster breakdown (no. of voxels)**
**Combined ALFF/fALFF and ReHo**					
**T2DM** **<** **control**					
R superior frontal gyrus, medial	6,60,8	−3.336	0.0004	76	R superior frontal gyrus, medial (69) L superior frontal gyrus, medial (5) R anterior cingulate/paracingulate gyri (1) L anterior cingulate/paracingulate gyri (1)
**ALFF/fALFF**					
**T2DM** **>** **control**					
R cerebellum, hemispheric lobule VIII	16,−74,−46	2.836	0.0023	48	R cerebellum, hemispheric lobule VIII (33) R cerebellum, hemispheric lobule VIIB (15)
**ReHo**					
**T2DM** **<** **control**					
R superior temporal gyrus	50,−2,−2	−2.900	0.0019	36	R superior temporal gyrus (17) R insula (17) R rolandic operculum (2)
L lingual gyrus	−2,−74,6	−3.166	0.0008	29	L lingual gyrus (16) L calcarine fissure/surrounding cortex (13)

*T2DM, type 2 diabetes mellitus; ALFF, amplitude of low-frequency fluctuations; fALFF, fractional ALFF; ReHo, regional homogeneity; MNI, Montreal Neurological Institute; SDM, seed-based d mapping; R, right; L, left*.

### Subgroup Analysis

Comparing with healthy controls, patients with T2DM showed significant regional hyperactivity in the right cerebellum in subgroup analysis of 10 ALFF/fALFF datasets, and hypoactivity in the left lingual gyrus extending to calcarine fissure/surrounding cortex and in the right superior temporal gyrus extending to the insula in the subgroup analysis of nine ReHo datasets (Table 2, Figure 2).

### Metaregression Analysis

**Figure 3:**
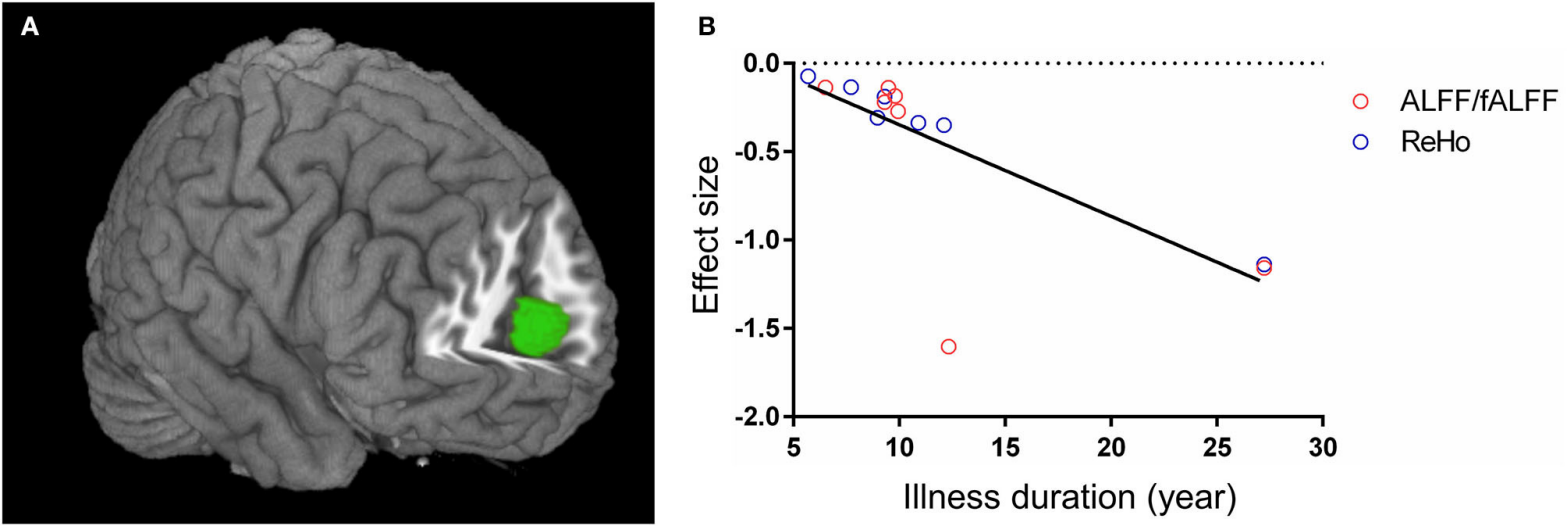
Metaregression analysis results. **(A)** The illness duration of patients with type 2 diabetes mellitus was negatively associated with regional activity in right anterior cingulate/paracingulate gyri, extending to bilateral medial superior frontal gyrus and left anterior cingulate/paracingulate gyri. **(B)** The effect sizes needed to create this plot were extracted from the peak voxel of maximum slope difference. The ALFF/fALFF and ReHo studies are represented as red and blue empty circles, respectively. The regression line (metaregression SDM slope) is presented as a straight line. ALFF, amplitude of low-frequency fluctuations; fALFF, fractional ALFF; ReHo, regional homogeneity; SDM, seed-based *d* mapping.

In the combined ALFF/fALFF and ReHo datasets, the whole-brain metaregression analysis found that the illness duration of patients with T2DM was negatively associated with regional activity in the right anterior cingulate/paracingulate gyri, extending to bilateral medial superior frontal gyrus and left anterior cingulate/paracingulate gyri (peak coordinates: *x* = 2, *y* = 54, *z* = 12, voxels = 255, *r* = 0.73, *p* = 0.0032). However, there were two outliers at the longest illness duration in the regression plot (Figure 3). When excluding the two datasets (Shi et al., 2020; Zhang Y. Q. et al., 2020), no significant association was found between the illness duration and regional activity in patients with T2DM. The mean age, percentage of males, education, onset age, HbA_1c_%, BMI, and MMSE scores were not linearly associated with regional function alteration in patients with T2DM.

When performing the whole-brain metaregression analysis for ALFF/fALFF and ReHo datasets separately, no significant associations were found between any regional function alterations and mean age, percentage of males, education, illness duration, onset age, HbA_1c_%, BMI, or MMSE.

### Heterogeneity and Publication Bias Analyses

All brain regions with functional alterations showed low between-study heterogeneity (*I*^2^ ranged from 1.60 to 19.04). The Egger test of funnel plot asymmetry did not identify any publication bias in any cluster (all *p* > 0.05). Detailed results of heterogeneity and publication bias analyses are provided in the Supplementary Material.

## Discussion

The present meta-analysis identified regional spontaneous neural activity alterations in patients with T2DM, including hypoactivity in the right medial superior frontal gyrus, right superior temporal gyrus, and left lingual gyrus, and hyperactivity in the right cerebellum. The novel findings of this study were hypoactivity in the medial superior frontal gyrus and hyperactivity in the cerebellum, which were inconsistent to previous meta-analysis (Xia et al., 2017).

The medial frontal cortex is a key part of the default mode network (DMN) and has been mainly implicated in value-based decision-making, regulation of negative emotion, self-perception, and social cognitive function (Bechara et al., 2000; Smith et al., 2009; Roy et al., 2012; Delgado et al., 2016; Hiser and Koenigs, 2018). The dysfunction of DMN was associated with the deficits in neurocognitive performance and episodic memory in patients with T2DM (Cui et al., 2015; Chen et al., 2016). We also identified negative correlation between regional activity in the medial superior frontal and anterior cingulate gyri and illness duration of patients with T2DM. This finding was consistent with previous studies that reported association between diabetic duration and structural and functional abnormalities in the medial pre-frontal cortex, such as gray matter atrophy (Garcia-Casares et al., 2014), white matter deficits (Hsu et al., 2012), decreased glucose metabolism (Garcia-Casares et al., 2014), and reduced functional connectivity (Liu et al., 2019). Besides, the hypoactivity in the medial pre-frontal cortex was found to correlate with diabetic microvascular disease (Wang et al., 2014), which was consistent with the observation of reduced cerebral blood flow in this region (Dai et al., 2017; Bangen et al., 2018). These findings suggested the functional alterations in the medial pre-frontal cortex might be the underlying pathophysiological mechanisms of cognitive deficits associated with cerebral small vessel disease in T2DM (Nelson et al., 2009; Umemura et al., 2013) and exacerbated with the progression of disease course (Korf et al., 2006). However, it should be noted that the correlation between regional function in the medial pre-frontal cortex and diabetic duration in our metaregression was not significant when excluding two outliers at long illness duration. As diabetic duration was found to be associated with the risk of cardiovascular autonomic neuropathy (Chen et al., 2008) and stroke (Banerjee et al., 2012), the longer disease duration might lead to the increased mortality (Maser et al., 2003; Pop-Busui et al., 2010). Thus, the potential survival bias might account for the lack of long diabetic duration sample. The robustness of association between regional function in the medial pre-frontal cortex and illness duration in T2DM needs to be further identified.

The cerebellum controls motor coordination and execution. Previous laboratory studies found the association between motor deficits and the cerebellar damage in diabetic rats (Sherin et al., 2010; Nagayach et al., 2014). The cerebellum dysfunctions also related to impaired cognition in patients with T2DM as structural and functional connectivity alterations were found in the cerebellar and cerebrocerebellar circuits (Fang et al., 2017; Zhang D. et al., 2020). The hyperactivity in the cerebellum might be a recruitment of additional neural resources to compensate for loss of cognitive function in the cerebral regions (Xia et al., 2013).

The regional hypoactivity in the left lingual gyrus extending to calcarine fissure/surrounding cortex and right superior temporal gyrus extending to insula was consistent with a previous meta-analysis (Xia et al., 2017). The lingual and calcarine surrounding gyri are key components of the visual cortex. Visuospatial dysfunction was common in T2DM (Moran et al., 2013), and the hypoactivity in the lingual gyrus was also correlated with impaired cognitive performance (Cui et al., 2014). The superior temporal gyrus is linked to language processing, which may be impaired by dysglycemia (Allen et al., 2015). The insula is an integral region in the salience network (Menon and Uddin, 2010), and hypoactivity may cause deficits in generation of appropriate behavioral responses to stimuli in patients with T2DM.

The present study showed some inconsistencies with previous meta-analysis (Xia et al., 2017). Our meta-analysis revealed hypoactivity in the right medial superior frontal gyrus and hyperactivity in the right hemispheric cerebellum, whereas previous meta-analysis revealed hyperactivity in the left medial superior frontal gyrus and hypoactivity in the right cerebellar culmen. There were several possible reasons accounting for the discrepancy. First, the former meta-analysis included only five original studies and seven datasets that investigated the regional functional activity alterations using ALFF/fALFF and ReHo. Our study with a larger sample included 16 original studies and 19 datasets, and many of the newly included studies reported hypoactivity in the medial superior frontal gyrus (Liu et al., 2016; Wang et al., 2019; Shi et al., 2020; Zhang Y. Q. et al., 2020) and hyperactivity in the cerebellum (Liao et al., 2019; Wang et al., 2019; Qi et al., 2020; Shi et al., 2020; Zhang Y. Q. et al., 2020). Second, the previous meta-analysis included both regional activity and functional connectivity measurements as well as perfusion modality, whereas this meta-analysis enrolled only studies using ALFF/fALFF and ReHo indices reflecting the regional spontaneous neural activity, avoiding heterogeneity of the included studies (Xia et al., 2017). Third, we performed this meta-analysis using a newly developed tool with nearly unbiased algorithm to estimate effect size (Albajes-Eizagirre et al., 2019b).

Several limitations should also be noted. First, as many original studies included patients with comorbidity, the effect of comorbidity on the regional functional activity alterations could not be assessed. Second, the combined analysis of ALFF/fALFF and ReHo datasets and subgroup analysis of ALFF/fALFF or ReHo datasets showed functional activity alterations in divergent regions. Thus, the different measurements might contribute to the heterogeneity of regional spontaneous neural dysfunction. Third, we detected relationship between functional activity alteration and diabetic duration; however, all the included studies were cross-sectional. Besides, this relationship was not significant when excluding two outliers at long illness duration. Thus, the metaregression result should be considered cautiously and further identified.

In conclusion, this meta-analysis identified concordant regional spontaneous neural activity abnormalities in patients with T2DM, characterized by hypoactivity in the medial pre-frontal cortex, visual cortex, and superior temporal gyrus, whereas hyperactivity in the cerebellum. These findings might represent the underlying neuropathological mechanisms of T2DM.

## Data Availability Statement

The original contributions presented in the study are included in the article/Supplementary Material, further inquiries can be directed to the corresponding author.

## Author Contributions

JL and PZ conceived and designed the study. JL, YL, XY, and HX collected the data. JL and YL analyzed the data and drafted the manuscript. All authors reviewed the manuscript and PZ revised the final manuscript. JL, JR, and PZ provided funding for the study. All authors contributed to the article and approved the submitted version.

## Conflict of Interest

The authors declare that the research was conducted in the absence of any commercial or financial relationships that could be construed as a potential conflict of interest.
