# Supplementary material for: Regional Spontaneous Neural Activity Alterations in Type 2 Diabetes Mellitus: A Meta-Analysis of Resting-State Functional MRI Studies
Source: Front Aging Neurosci. 2021 Jun 17;13:678359. doi: 10.3389/fnagi.2021.678359 (PMC8245688; doi:10.3389/fnagi.2021.678359)
Supplement: Supplementary file 1 [file Data_Sheet_1.DOCX]

***Supplementary Material***

**TABLE S1.** Methodology and acquisition parameters for studies included in meta-analysis.

| **Study (year)** | **Methodology** | **Scanner** | **Resolution (mm)** | **Software** | **FWHM (mm)** | **Threshold** |
| --- | --- | --- | --- | --- | --- | --- |
| Xia (2013) | ALFF | 3T Siemens Trio | 3.75×3.75×4 | SPM8 | 4 | AlphaSim: p<0.05 corrected |
| Cui (2014) | ALFF/ReHo | 3T Siemens Trio | thickness 4 | SPM8 | 4 | AlphaSim: p<0.01 corrected |
| Wang (2014) | ALFF | 3T Phillips Achieva | 3×3×5 | SPM8 | 4 | AlphaSim: p<0.05 corrected |
| Zhou (2014) | ALFF | 3T Siemens | 4×4×4 | SPM5 | 8 | p<0.05 corrected |
| Liu (2016) | ReHo | 3T Siemens Trio | 3×3×3 | SPM8 | 4 | AlphaSim: p<0.01 corrected |
| Peng (2016) | ReHo | 3T GE Signa Hdxt | 3.75×3.75×4 | SPM8 | 4 | AlphaSim: p<0.05 corrected |
| Wang (2017a) | fALFF | 3T Siemens Trio | 3.75×3.75×4 | SPM8 | 6 | AlphaSim: p<0.05 corrected |
| Wang (2017b) | ALFF | 3T Phillips | 1.8×1.8×4 | SPM8 | 8 | AlphaSim: p<0.01 corrected |
| Liao (2019) | ReHo | 3T Siemens Trio | 3.4×3.4×4 | SPM8 | 4 | AlphaSim: p<0.01 corrected |
| Wang (2019) | ALFF | 3T Siemens Trio | 3.4×3.4×4 | SPM | 6 | AlphaSim: p<0.01 corrected |
| Liu (2020a) | ALFF | 3T Siemens Trio | 3×3×3 | SPM | 4 | Gaussian random field: p<0.05 corrected |
| Liu (2020b) | ReHo | 3T GE | 3.75×3.75×3 | SPM8 | 8 | AlphaSim: p<0.01 corrected |
| Qi (2020) | ALFF | 3T GE Discovery 750 | 3.75×3.75×3 | SPM8 | NA | Gaussian random field: p<0.05 corrected |
| Xiong (2020) | ReHo | 3T GE Discovery 750 | 3.75×3.75×4 | SPM12 | NA | AlphaSim: p<0.01 corrected |
| Shi (2020) | ALFF | 3T Siemens Trio | 3.4×3.4×4 | SPM | 6 | AlphaSim: p<0.01 corrected |
| Zhang (2020b) | ReHo | 3T Siemens Trio | 3.4×3.4×4 | SPM8 | 6 | AlphaSim: p<0.01 corrected |

ALFF, amplitude of low-frequency fluctuations; fALFF, fractional ALFF; ReHo, regional homogeneity; SPM, Statistical Parametric Mapping; FWHM, full width at half maximum.

**TABLE S2.** Results of heterogeneity analysis.

| **Brian areas** | **MNI coordinates** | ***I^2^* (%)** |
| --- | --- | --- |
| **Combined ALFF/fALFF and ReHo** |  |  |
| R superior frontal gyrus, medial | 6,60,8 | 19.04 |
| **ALFF/fALFF** |  |  |
| R cerebellum, hemispheric lobule VIII | 16,-74,-46 | 9.10 |
| **ReHo** |  |  |
| R superior temporal gyrus | 50,-2,-2 | 3.34 |
| L lingual gyrus | -2,-74,6 | 1.60 |

MNI, Montreal Neurological Institute; ALFF, amplitude of low-frequency fluctuations; fALFF, fractional ALFF; ReHo, regional homogeneity; R, right; L, left.

**TABLE S3.** Results of the funnel plots and Egger test.

| **Brian areas** | **MNI coordinates** | **Funnel plots** | **Eager test** |
| --- | --- | --- | --- |
| **Combined ALFF/fALFF and ReHo** |  |  |  |
| R superior frontal gyrus, medial | 6,60,8 | 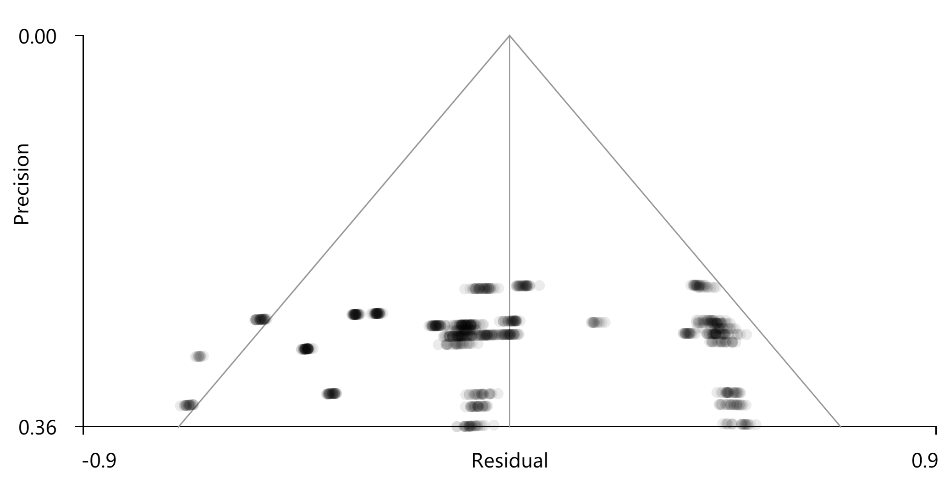 | *Z*= -0.13, *p* = 0.89 |
| **ALFF/fALFF** |  |  |  |
| R cerebellum, hemispheric lobule VIII | 16,-74,-46 | 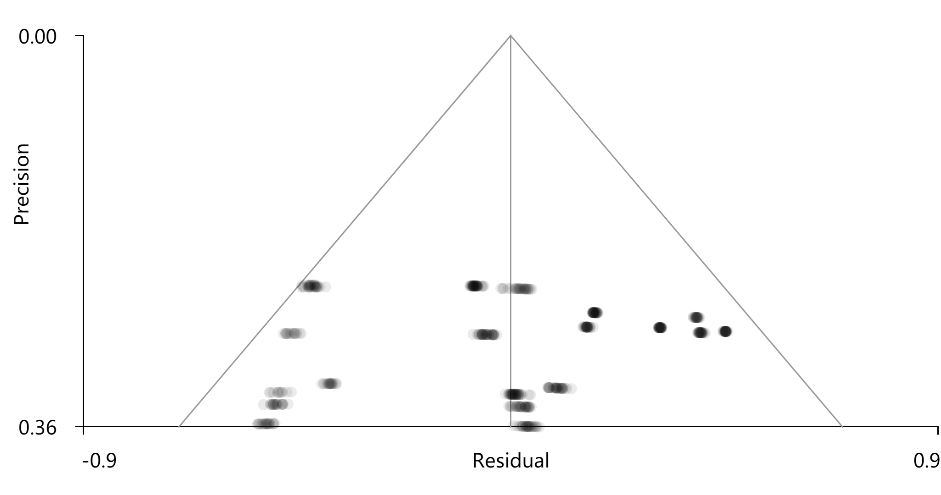 | *Z*= -0.33, *p* = 0.74 |
| **ReHo** |  |  |  |
| R superior temporal gyrus | 50,-2,-2 | 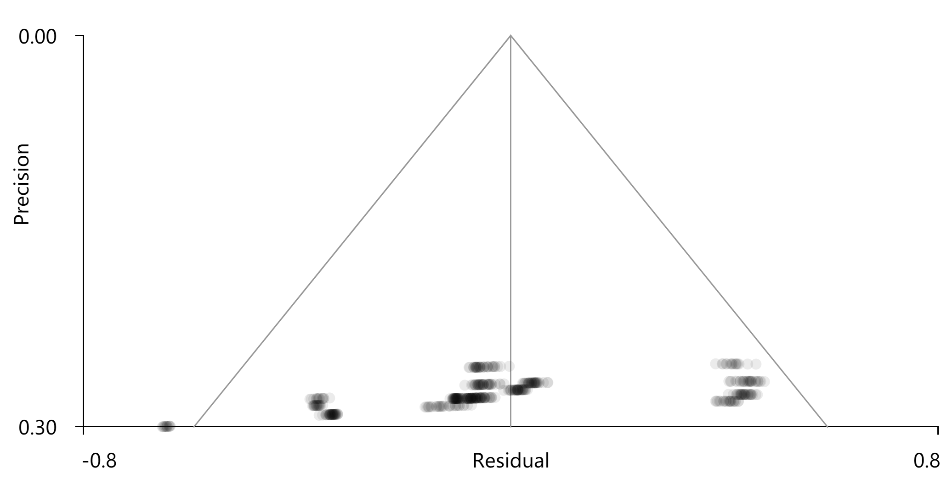 | *Z*= -1.16, *p* = 0.24 |
| L lingual gyrus | -2,-74,6 | 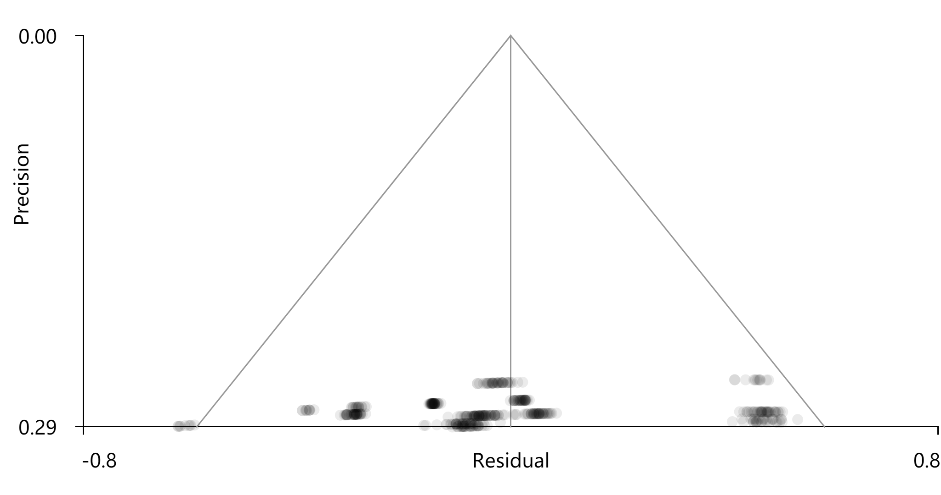 | *Z*= -0.15, *p* = 0.88 |

MNI, Montreal Neurological Institute; ALFF, amplitude of low-frequency fluctuations; fALFF, fractional ALFF; ReHo, regional homogeneity; R, right; L, left.
